# A Repeat Sequence Causes Competition of ColE1-Type Plasmids

**DOI:** 10.1371/journal.pone.0061668

**Published:** 2013-04-16

**Authors:** Mei-Hui Lin, Jen-Fen Fu, Shih-Tung Liu

**Affiliations:** 1 Department of Medical Biotechnology and Laboratory Science, College of Medicine, Chang Gung University, Taoyuan, Taiwan; 2 Department of Medical Research, Chang Gung Memorial Hospital, and Graduate Institute of Clinical Medical Sciences Taoyuan, Taiwan; 3 Department of Microbiology and Immunology, College of Medicine, Chang Gung University, Taoyuan, Taiwan; 4 Research Center for Pathogenic Bacteria, Chang Gung University, Taoyuan, Taiwan; Florida International University, United States of America

## Abstract

Plasmid pSW200 from *Pantoea stewartii* contains 41 copies of 15-bp repeats and has a replicon that is homologous to that of ColE1. Although deleting the repeats (pSW207) does not change the copy number and stability of the plasmid. The plasmid becomes unstable and is rapidly lost from the host when a homoplasmid with the repeats (pSW201) is present. Deleting the repeats is found to reduce the transcriptional activity of *RNAIp* and *RNAIIp* by about 30%, indicating that the repeats promote the transcription of *RNAI* and *RNAII,* and how the RNAI that is synthesized by pSW201 inhibits the replication of pSW207. The immunoblot analysis herein demonstrates that RNA polymerase β subunit and σ^70^ in the lysate from *Escherichia coli* MG1655 bind to a biotin-labeled DNA probe that contains the entire sequence of the repeat region. Electrophoretic mobility shift assay also reveals that purified RNA polymerase shifts a DNA probe that contains four copies of the repeats. These results thus obtained reveal that RNA polymerase holoenzyme binds to the repeats. The repeats also exchange RNA polymerase with *RNAIp* and *RNAIIp in vitro*, revealing the mechanism by which the transcription is promoted. This investigation elucidates a mechanism by which a plasmid prevents the invasion of an incompatible plasmid and maintains its stability in the host cell during evolution.

## Introduction

Stability is the most important determinant of the survival of a plasmid during evolution [1]. To maintain stability, a plasmid with a low copy number, such as F or P1, uses complex segregation systems to ensure it is precisely segregated into daughter cells following plasmid replication and cell division [2], [3], [4]. Post-segregational killing (PSK) systems also ensure that all of the living daughter cells receive a plasmid following cell division [5]. Many plasmids with a relatively high copy number, including ColE1 and p15A, typically segregate random numbers of replicated plasmids [6], [7]. Since the number of copies is large, the probability that a daughter cell does not receive a randomly segregated plasmid is low [7], [8], [9]. Additionally, precise control of the copy number ensures that a constant number of plasmids are maintained in a cell [6], [10]. Incompatibility is another factor that is likely to threaten the stability of a plasmid [11]. After an incompatible plasmid invades a cell, the plasmid coexists with the resident plasmid and consumes the same resources in the cell to replicate and segregate. Over time, the plasmid that invades the cell may multiply and cause the loss of the original resident plasmid.

Plasmid competition is a mechanism by which a plasmid is lost in the presence of an incompatible plasmid in the same cell. One of the best-documented instances of this phenomenon concerns pT181. This plasmid contains a *cis* element, *cmp*, which functions at a distance and optimizes the utilization of *oriV* by a replication initiation protein, RepC [12], [13]. Gennaro *et al.* [14], [15] showed that although deleting *cmp* from pT181 does not affect a plasmid’s replication, stability, or copy number, a homoplasmid without a *cmp* is rapidly lost when pT181 is present in the same cell [14], [15]. Plasmid competition also occurs in pSC101 and is associated with a DNA sequence that involves plasmid partition [16]. If pSC101 is present, then a homoplasmid without this sequence is lost from the cell [17]. Additionally, PSK-mediated plasmid competition has been identified. A plasmid that encodes a PSK system can cause the loss of a PSK-negative plasmid from the same cell [18], [19], [20].

Plasmids of the ColE1 family replicate by a different mechanism from that used by pT181 and pSC101. Rather than using replication initiation proteins to initiate plasmid replication, ColE1 synthesizes preprimer RNA [21], [22], which forms an RNA-DNA hybrid at the *oriV*, allowing cleavage of the RNA by RNase H to produce RNAII [23], [24], which then serves as a primer to initiate plasmid replication [25], [26], [27]. Therefore, RNAII acts only in *cis* and cannot initiate the replication of another ColE1 molecule. Also, ColE1 synthesizes RNAI, which forms a duplex with the 5′ region of preprimer RNA [27]; this duplex changes the structure of the preprimer RNA, preventing its cleavage at the *oriV* and, thereby, the initiation of plasmid replication [27], [28]. Therefore, RNAI is a *trans*-acting factor that acts as an *inc* determinant that controls the copy number and causes plasmid incompatibility [29], [30].

**Figure 1:**
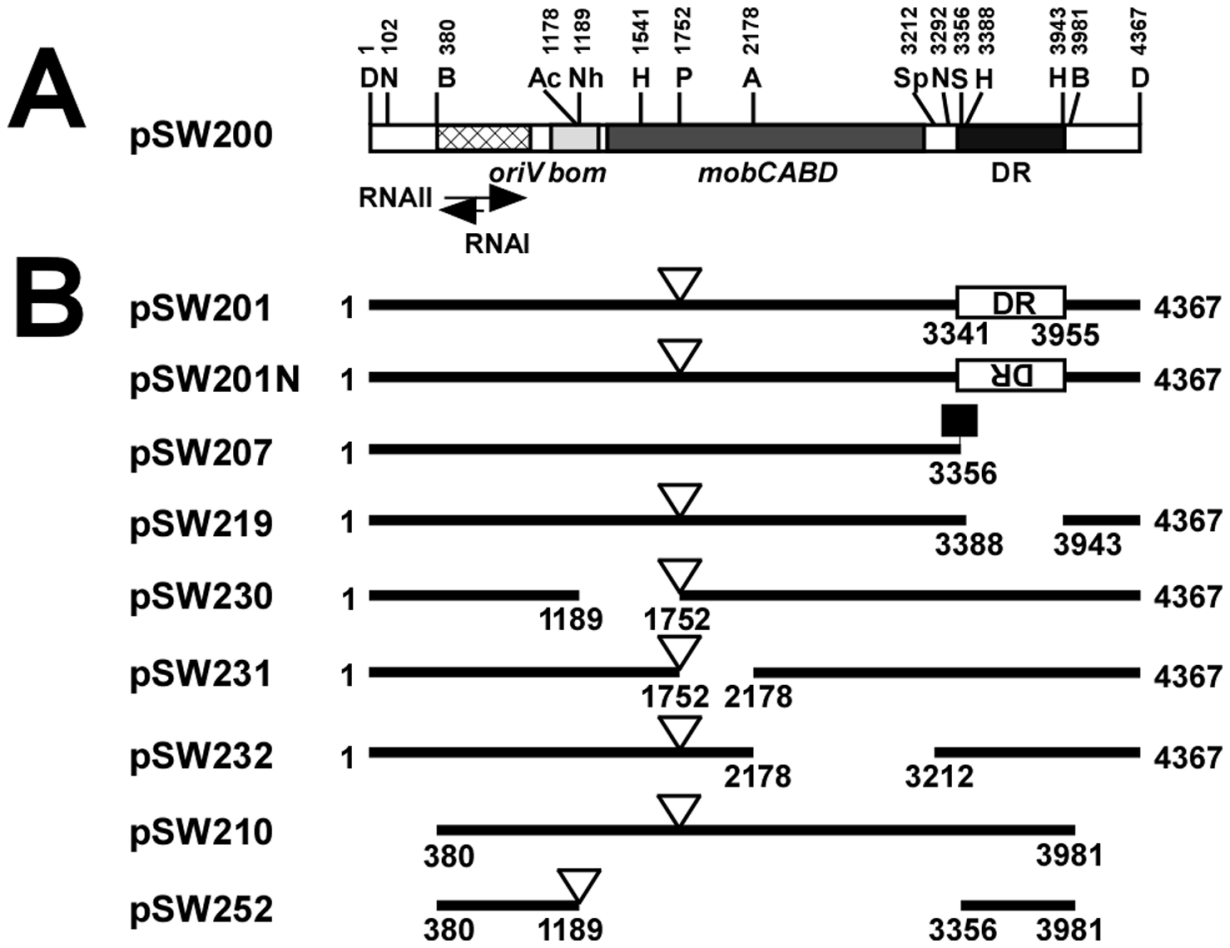
Linear map of plasmids used in this study. (A) Plasmid pSW200 (4367 bp) has an *RNAI*-*RNAII* region, *oriV*, *bom*, *mobCABD* and a region that contains 41 copies of 15-bp direct repeats (DR). (B) Plasmid pSW201 includes the entire pSW200 sequence and a Km-resistance gene (open triangle) that is inserted into the *mob* region at a *Pst*I site. Plasmid pSW201N is identical to pSW201 except for the inverted DR region. Plasmids pSW210, pSW219, pSW230, pSW231, pSW232 and pSW252 are deletion derivatives of pSW201. Plasmid pSW207 contains 40 of the 41 repeats with the *Ssp*I-*Dra*I fragment replaced by a Tc-resistance gene (filled square). Numbers represent the nucleotide positions from the *Dra*I site in pSW200. The map shows the following restriction enzyme sites; A, *Afl*III; B, *Bgl*II; D, *Dra*I; H, *Hinc*II; N, *Nco*I; Ac, *Acc*I; Nh, *Nhe*I; P, *Pst*I; S, *Ssp*I, and Sp, *Spe*I.

*Pantoea stewartii* SW2 is a corn pathogen that induces necrosis and systemic wilting, called Stewart’s wilt [31]. The organism has 13 plasmids, which range in size from 4 to 320 kb [32]. Since *P. stewartii* is a member of Enterobacteriaceae, these plasmids can replicate and be stably maintained in *Escherichia coli* [33]. Of these 13 plasmids in strain SW2, pSW100 (4272 bp) and pSW200 (4367 bp) are the two smallest and their replicon is homologous to those of ColE1 and p15A [34], [35]. However, in spite of the homology of the replicon sequence, the two plasmids are compatible with ColE1 and p15A [34], [35]. Interestingly, pSW100 and pSW200 have only ten copies per cell – fewer than ColE1 [34], [35]. A calculation that is based on their copy number shows that the plasmids are less than one thousandth as stable as ColE1 or p15A [6], [36]. Owing to their potential instability, pSW100 uses the sex pilus assembly as a partition tool to maintain its stability whereas pSW200 utilizes a 9-bp sequence, *sps* (sequence for plasmid stability) for the efficient synthesis of RNAII and maintenance of the stability of the plasmid [37], [38]. Plasmid pSW200 also contains genes that are required for plasmid mobilization and a 0.6-kb fragment, from nt 3341 to nt 3955, which includes 41 copies of contiguous 15-bp repeats (DR region) (Fig. 1). An earlier study revealed that although deleting 40 of the 41 repeats from pSW200 (pSW207) does not affect the stability or copy number of the plasmid [35], the plasmid becomes extremely unstable when a homoplasmid with an intact DR region, pSW201, is present in the same cell [35]. The loss of pSW207 is associated with the DR region in pSW201 because another pSW201 derivative that lacks the DR region, pSW219, is incompatible with pSW207 and does not unilaterally cause the loss of pSW207 [35]. This investigation finds that the repeats promote the transcription of both the *RNAI* and *preprimer RNA* genes. The elevated RNAI expression from pSW201 prevents preprimer RNA from coupling to the *oriV* in pSW207, inhibiting replication and resulting in the loss of pSW207.

## Materials and Methods

### Bacterial Strains and Culturing Conditions

*E. coli* HB101 (F^-^, *hsdS20, supE44, recA13, ara14, proA2, rpsL20, xyl-5, mtl-1*) [39] was used as a host for studying competition among plasmids. *E. coli* MG1655 (F^-^, λ^-^) [40] was obtained from Bioresource Collection and Research Center (Taiwan) and used to identify the proteins that bind to DR repeats. LB broth and agar [41] were used as general-purpose media. Kanamycin (Km) (50 µg/ml), ampicillin (Ap) (50 µg/ml), and tetracycline (Tc) (12.5 µg/ml) were added to the medium to select antibiotic-resistant colonies.

### Plasmid Construction

Plasmids that were used in this study are summarized in Table 1 and shown in Fig. 1. The construction of pSW201, pSW207, pSW210, pSW219 and pSW106 has been described elsewhere [35]. Plasmid pSW201N is identical to pSW201 except for the opposite orientation of the DR region. One to four 0.6-kb fragments from pACYC184 (nt 3808 to nt 162, GenBank accession number: X06403.1) [42] were inserted into *Dra*I site in pSW201 to produce pSW201D1, pSW201D2, pSW201D3 and pSW201D4, respectively, to study the effects of the distance between the 15-bp repeats and the *RNAI* and *RNAII* promoters on competition. Plasmids pSW230, pSW231 and pSW232 are derivatives of pSW201 that lack the *Nhe*I-*Pst*I (nt 1189 to nt 1752), *Pst*I-*Afl*III (nt 1752 to nt 2178), and *Afl*III-*Spe*I fragments (nt 2178 to nt 3212), respectively, of pSW200. Plasmid pSW252 contains the *Bgl*II-*Nhe*I fragment (nt 380 to nt 1189) and the *Ssp*I-*Bgl*II fragment (nt 3356 to nt 3981) in pSW200, and a kanamycin-resistance gene. A *Bsp*HI-*Acc*I fragment, a *Bgl*II-*Acc*I fragment, a *Bst*I-*Acc*I fragment and a *Bgl*II-*Acc*I fragment, which contained the minimal replicons of pBR322 [43], pACYC184 [42], pSW100 [34] and pRK415 [44], were isolated by restriction digestion and used to replace the *Bgl*II-*Acc*I fragment in pSW200 to form pBR322-210, pACYC-210, pSW100-210 and pRK-210, respectively. Plasmid pKK175-6-lux was constructed by inserting a *Hin*dIII-*Bam*HI fragment of pUCD1752, which contains the *luxAB* genes, into the *Hin*dIII-*Bam*HI sites of pKK175-6 [45]. Then, a DNA fragment that covered the region from nt 1 to 426 and nt 3356 to 4367 in pSW200 were inserted into pKK175-6-lux to form pSW242 and pSW243, respectively. PCR was used to amplify a DNA fragment from nt 3022 to nt 426, which contains the DR region and *RNAIIp,* with primers A1 (5′-CCCTGCAGATGACGGAGCTGGAAAAAC) and B1 (5′-GGAAGCTTCAGTTAATAAGATTACGGCGGG) using pSW210 as a template. The PCR product was cut using *Pvu*II and *Hin*dIII and inserted into the *Sma*I-*Hin*dIII sites in pKK175-6 [45] to form pSW261. The DR region in pSW261 was deleted using *Nco*I and *Bgl*II to form pSW262. The region from nt 532 to nt 957 that contained *RNAIp* in pSW200 was amplified by PCR using primers A2 (5′-GGAGGATCCTTCCAGTGTAGCCGCAG) and B2 (5′-CCGTCGACGCTGCGATGCCGTTTTTCC) and inserted into the *Bam*HI-*Sal*I sites in pSW261 to yield pSW2611. A DNA fragment that contained a 5′ portion of the *tet* gene was amplified by PCR using pKK175-6 as a template and primers A3 (5′-CCAAGCTTCCTAATGAGGAGTCGCATAA) and B3 (5′-CCCGGATCCATAAGTGCGCGACGATAGT). The fragment was inserted at the *Bam*HI-*Hin*dIII sites in pSW2611 to form pSW2612. *Nco*I and *Bgl*II were used to delete *RNAIIp* from pSW2612 to form pSW263. The *Nco*I-*Hin*dIII fragment, which contained the DR region and *RNAIIp* in pSW2612, was then deleted to generate pSW264. *Aat*II and *Spe*I were used to delete the DR region in pSW201, which was then treated with ExoIII and mungbean nuclease using an Exo-size deletion kit (New England Biolabs). The DNA fragments were then ligated to generate plasmids with various numbers of repeats. DNA sequencing was performed to count the repeats in the plasmids.

**Table 1:** Plasmids used in this study.

Plasmid	Characteristics	Reference
pSW201	A derivative of pSW200 containing a Km-resistance gene inserted at the *Pst*I site; containing DR	35
pSW207	A plasmid containing the *Dra*I*-Ssp*I fragment of pSW200 and a Tc-resistance gene; without DR	35
pSW210	A derivative of pSW201 that lacks the *Bgl*II fragment (nt 3981 to nt 380), the region between DR and *RNAII*;containing DR	35
pSW219	A derivative of pSW201 that lacks the *Hin*cII fragment (nt 3388 to nt 3943); without DR	35
pSW106	A plasmid containing the *Ssp*I fragment of pSW100 and an Ap-resistance gene	34
pSW201N	A plasmid identical to pSW201 except that the DR region is oppositely orientated; containing DR	This study
pSW201D1	A derivative of pSW201 with one copy of 0.6-kb fragment from pACYC184 (nt 3808 to nt 162) inserted atthe *Dra*I site; containing DR	This study
pSW201D2	A derivative of pSW201 with two copies of 0.6-kb fragment from pACYC184 (nt 3808 to nt 162) inserted atthe *Dra*I site; containing DR	This study
pSW201D3	A derivative of pSW201 with three copies of 0.6-kb fragment from pACYC184 (nt 3808 to nt 162) inserted atthe *Dra*I site; containing DR	This study
pSW201D4	A derivative of pSW201 with four copies of 0.6-kb fragment from pACYC184 (nt 3808 to nt 162) inserted atthe *Dra*I site; containing DR	This study
pSW230	A derivative of pSW201 that lacks the *Nhe*I-*Pst*I fragment (nt 1189 to nt 1752); *mobC* deleted; containing DR	This study
pSW231	A derivative of pSW201 that lacks the *Pst*I-*Afl*III fragment (nt 1752 to nt 2178); *mobA* deleted; containing DR	This study
pSW232	A derivative of pSW201 that lacks the *Afl*III-*Spe*I fragment (nt 2178 to nt 3212); *mobABD* deleted; containing DR	This study
pSW252	A plasmid consisting of the *Bgl*II-*Nhe*I fragment (nt 380 to nt 1189) and *Ssp*I-*Bgl*II fragment (nt 3356 to nt 3981)of pSW200 and a Km-resistance gene; containing DR	This study
pBR322-210	A pSW210 derivative with its *Bgl*II-*Acc*I fragment replaced by a *Bsp*HI-*Acc*I fragment from pBR322, whichcontains the replicon of ColE1; containing DR	This study
pACYC-210	A pSW210 derivative with its *Bgl*II-*Acc*I fragment replaced by a *Bgl*II-*Acc*I fragment from pACYC184,which contains the minimal replicon of p15A; containing DR	This study
pSW100-210	A pSW210 derivative with its *Bgl*II-*Acc*I fragment replaced by a *Bst*I-*Acc*I fragment from pSW106, whichcontains the minimal replicon of pSW100; containing DR	This study
pRK-210	A pSW210 derivative with its *Bgl*II-*Acc*I fragment replaced by a *Bst*I-*Acc*I fragment from pRK415, whichcontains the minimal replicon of pRK415; containing DR	This study
pKK175-6-lux	A pKK175-6 derivative with a *Hin*dIII-*Bam*HI fragment of pUCD1752, which contained the *luxAB* genes, insertedat the *Hin*dIII-*Bam*HI sites; a fusion vector	This study
pSW242	A transcriptional fusion between *RNAIIp* and *luxAB*; a DNA fragment from nt 1 to 426 in pSW200 inserted inpKK175-6-lux; without DR	This study
pSW243	A DNA fragment from nt 3356 to 4367 in pSW200 inserted in pKK175-6-lux; containing DR	This study
pSW261	A transcriptional fusion between *RNAIIp* and tetracycline-resistance gene for the analysis of promoter activityby RT-qPCR; a DNA fragment from nt 3022 to nt 426 in pSW210 inserted in pKK175-6; containing DR	This study
pSW262	Same as pSW261 but without DR	This study
pSW263	A transcriptional fusion between *RNAIp* and a fragment from the tetracycline-resistance gene for the analysisof promoter activity by RT-qPCR; a DNA fragment from nt 957 to nt 3822 in pSW200 inserted inpKK175-6; containing DR	This study
pSW264	Same as pSW263 but without DR	This study

### Competition Assay

*E. coli* HB101 was cotransformed with pSW207 (Tc^r^) and a deletion derivative of pSW201 (Km^r^). Transformants were cultured overnight in LB broth that contained Km and Tc, and subcultured to the mid-log phase in the same medium without antibiotics. Plasmids in the cell were analyzed using the alkaline-lysis method of Kado and Liu [46] with minor modification. Briefly, the plasmids were extracted from cells (1×10^9 ^cfu). The cell pellet was dissolved in 20 µl TAE and 80 µl extraction buffer [46]. After 1 hr of incubation at room temperature, the plasmid solution was treated with 100 µl phenol-chloroform. Meanwhile, cells (2×10^5^ cfu) were subcultured in 5 ml of antibiotic-free LB broth for 7 h with shaking at 37°C, plated on LB agar, and then replica-plated onto plates that contained Km or Tc. About 500 to 1000 colonies were examined to determine the fraction of the colonies that were resistant to kanamycin and tetracycline. Cells that had been transformed using a single plasmid were used as a control. The ratio of copy number of two plasmids in the same cell was estimated following agarose gel electrophoresis by measuring the intensity of the plasmid bands using the Gel-Pro software program (Media Cybernetics). Plasmid pSW201, which has ten copies per cell [35], was used as a reference.

### DNA Affinity Precipitation Assay (DAPA)

*E. coli* MG1655 was cultured in 50 ml LB overnight. After centrifugation, cell pellet was suspended in ice-cold homogenization buffer (50 mM NaH_2_PO_4_, 300 mM NaCl, 10 mM imidazole, 1.5% Triton X-100, pH 8.0). Cells were homogenized using a Mini BeadBeater (Biospec Products Inc.). The cell lysate was centrifuged at 17,000×g for 30 min at 4°C. A biotinylated DNA probe, DR-I, which contains the entire sequence of the DR region (nt 3314 to nt 3975) in pSW200, was amplified by PCR using biotin-labeled primers 200-Bio-F5 (5′- GTTAGTCCCTTCCACATTAA) and 200-R5 (5′-ACGATGGGGTTATCAATCTG). The lysate (1 mg protein) was mixed with 2.5 µg of DR-I probe in a binding buffer that contained 60 mM KCl, 12 mM HEPES, pH 7.9, 4 mM Tris-HCl, 5% glycerol, 0.5 mM EDTA, and 1 mM dithiothreitol. The reaction mixture was incubated on ice for 45 minutes and mixed with 30 mg M280 streptavidin beads (Dynal Biotech, Norway). The beads were then captured using a magnet and washed five times in binding buffer while they were attached to the magnet. A 2× electrophoresis sample buffer was used to extract proteins that were bound to the probes, and the proteins were then boiled for 10 min [37]. The proteins were separated by in a 6% SDS-polyacrylamide gel and analyzed by immunoblotting using anti-RNA polymerase β subunit antibody and anti-σ^70^ antibody (Abcam, Cambridge, UK) [37]. A biotin-labeled probe, 167, which contained a non-promoter sequence from the *fen* operon [47] was used as a negative control.

### Electrophoretic Mobility Shift Assay (EMSA)

Biotin was used to label double-stranded DNA probes using a 3′-end DNA labeling kit (Pierce, Rockford, IL). Each probe (5 nM) was then mixed with RNA polymerase holoenzyme (Epicentre, Madison, WI) in 20 µl of a reaction mixture that contained 1 mM Tris-HCl, pH 7.5, 50 mM KCl, 1 mM dithiothreitol, 2.5% glycerol, 5 mM MgCl_2_, 1 µg poly(dI-dC), and 0.05% NP-40. The mixture was incubated at room temperature for 20 min. EMSA was performed using the method of Lin *et al*. [37]. An unlabeled DR probe was used to compete the binding. DNA was detected using horseradish peroxidase-conjugated streptavidin (Pierce, Rockford, IL). DNA probes were also labeled using [γ-^32^P]ATP according to a method that has been described elsewhere [48]. ^32^P-labeled DNA was then purified using an Easy Pure PCR/Gel Extraction Kit (Bioman, Taipei, Taiwan). RNA polymerase (5 nM) was incubated with ^32^P-labeled DR probe (0.1 µM) in 20 µl EMSA reaction mixture. After 20 min of incubation, unlabeled 167, *RNAIp* and *RNAIIp* probes (0.1 µM) were added to the mixture to detect the exchange of RNA polymerase from the DR probe to 167, *RNAIp* and *RNAIIp* probes. In another set of exchange experiments, RNA polymerase (5 nM) was incubated with biotin-labeled DR probe (1 nM) for 15 min. The DNA-protein complex was captured using the method that was used for DAPA. ^32^P-labeled 167, *RNAIp* and *RNAIIp* probes (0.1 µM) were then added to the DNA-protein complex. Exchange of RNA polymerase from the DR probes to *RNAIp* and *RNAIIp* was analyzed by gel electrophoresis and autoradiography.

### Analysis of Transcription Using RT-qPCR

An RNA/DNA Mini Kit (Qiagen, Valencia, CA) was used to purify RNA from *E. coli* cells that had been cultured to the log phase. Purified RNA was suspended in water that had been treated with diethyl pyrocarbonate and reverse-transcribed to cDNA using both random hexamers and Superscript II reverse transcriptase (Life Technologies). An ABI PRISM 7700 sequence detection system (Applied Biosystems) was used to quantify and analyze the cDNA. 5′-TCCTAATGCAGGAGTCGCATAA and 5′-CATAAGTGCGGCGACGATAGT were used in PCR to amplify a 115-bp region in the tetracycline-resistance gene. The 16S rRNA, which was used as an internal control to normalize the amount of tested transcript, was reverse-transcribed and amplified using primers F1 (5′-CCATGAAGTCGGAATCGCTAG) and R1 (5′-ACTCCCATGGTGTGACGG) [49]. The activities of *RNAIp* and *RNAIIp* were normalized by calculating the difference between the threshold number of cycles of 16S rRNA and that of the tetracycline-resistance gene (ΔCt). The difference between the promoter activity in the presence of DR and that in the absence of DR was determined from the difference between ΔCt values (ΔΔCt). The amount of *tet* mRNA that was obtained from pSW261 and pSW263 was set to 100%. The difference between the amounts of mRNA was calculated as 2^ΔΔCt^ [50]. Each experiment was performed three times.

## Results

### Repeat Region and Plasmid Competition

Our earlier study demonstrated that the repeats in pSW201 are responsible for the instability of pSW207 (Fig. 1B) as a pSW201 derivative that does not include an intact repeat region, such as pSW219, cannot destabilize pSW207 [35] (Table 2). This study further demonstrated that pSW207 was lost in the presence of pSW252, in which all of the regions other than those that contained the replicon and repeats of pSW200 were deleted (Fig. 1B). After *E. coli* HB101 was cotransformed with pSW252 and pSW207, 94% of the colonies were found to be resistant to kanamycin (pSW252), and 6% were resistant to tetracycline (pSW207) (Table 2), verifying that the repeats in pSW252 are responsible for plasmid competition. Additionally, pSW207 is lost irrespectively of the orientation of the DR region in pSW201 because pSW201N, which contains an inverted DR region (Fig. 1B), caused the loss of pSW207 with a pSW201N:pSW207 segregation ratio of 98∶2 (Table 2). Furthermore, increasing the distance between DR and the *RNAII* promoter by inserting one to four copies of a 600-bp DNA fragment at a *Dra*I site to form pSW201D1, pSW201D2, pSW201D3 and pSW201D4, respectively, did not influence the capacity of pSW201 to destabilize pSW207, all of which had a segregation ratio with pSW207 of 99∶1 (Table 2). In this study, the *mob* region was also deleted (pSW230, pSW231, pSW232) (Fig. 1B). These plasmids destabilized pSW207 (Table 2), suggesting that the *mob* genes are not associated with competition.

**Table 2:** Exclusion of pSW207 by pSW201 derivatives.

Plasmid A	Plasmid B	Colony Ratio	Plasmid Ratio	DR in A	
(Km^r^)	(Tc^r^)	(A:B)	(A:B)		
pSW201	pSW207	97∶3	10∶1	+	
pSW219	pSW207	55∶45	1∶1	–	
pSW252	pSW207	94∶6	10∶1	+	
pSW201N	pSW207	98∶2	10∶1	+	
pSW201D1	pSW207	99∶1	10∶1	+	
pSW201D2	pSW207	99∶1	10∶1	+	
pSW201D3	pSW207	99∶1	10∶1	+	
pSW201D4	pSW207	99∶1	10∶1	+	
pSW230	pSW207	99∶1	10∶1	+	
pSW231	pSW207	96∶4	10∶1	+	
pSW232	pSW207	94∶6	10∶1	+	

### Numbers of Repeats and Competition Among Plasmids

**Figure 2:**
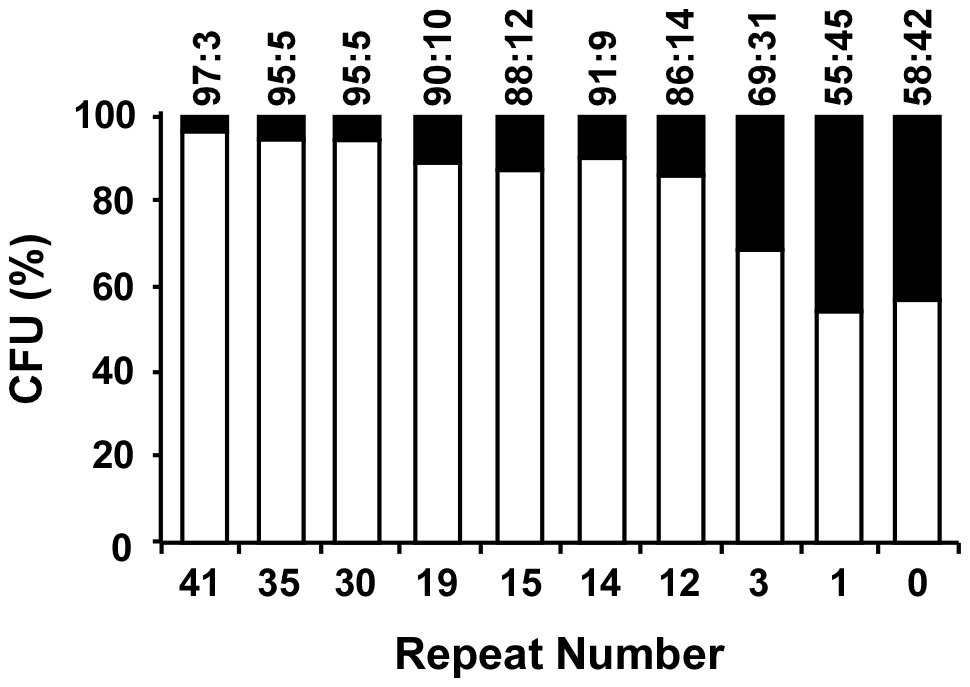
Correlation between number of repeats and competition among plasmids. Derivatives of pSW201 that contain various numbers of 15-bp repeats were cotransformed with pSW207 into *E. coli* HB101. The transformants were plated on LB agar and then replica-plated on LB agar that contained Km or Tc to select those that contained pSW201 derivatives with various numbers of repeats (empty column) and pSW207 (filled column).

Since the 15-bp repeats in pSW200 importantly affected plasmid competition, whether altering the number of the repeats could influence the capacity of pSW201 to compete with pSW207 was studied. The competition assay revealed that only 3% of the population contained pSW207 when the cells were cotransformed with pSW201. The fraction of the cells that contained pSW207 increased to 10% when the number of repeats in pSW201 was reduced from 41 to 19; it increased to 31%, 45% and 42% when the number of repeats in pSW201 was reduced to three, one and zero, respectively (Fig. 2). The results demonstrate that number of repeats strongly affects plasmid competition.

### Enhancing Transcriptional Activity of *RNAIp* and *RNAIIp* by 15-bp Repeats

**Figure 3:**
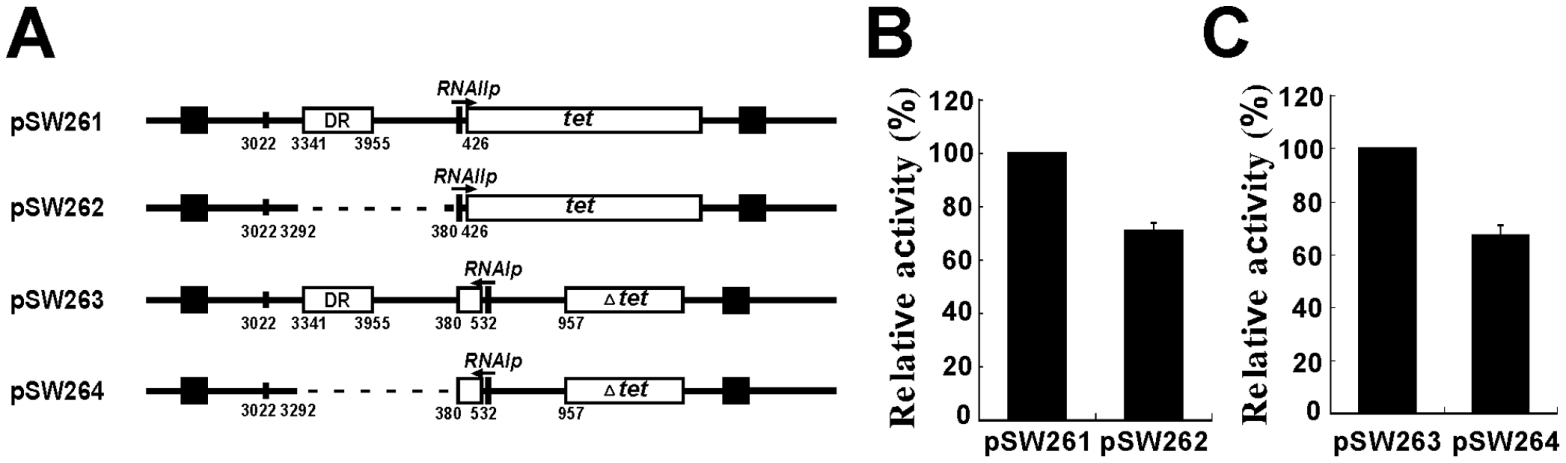
RT-qPCR analysis of activity of *RNAIp* and *RNAIIp*. (A) Map of pSW261, pSW262, pSW263, and pSW264. Arrow indicates direction of transcription. Numbers represent relative nucleotide positions in pSW200. (B)(C) RNA that was transcribed from a region in Tc-resistance gene was amplified by RT-qPCR. 16S rRNA was used as an internal control and amounts of mRNA that were transcribed from *RNAIp* and *RNAIIp* were normalized to amount of 16S rRNA. Amount of *tet* mRNA from pSW261 and pSW263 was set to 100%. Experiment was performed three times and each sample was prepared in duplicate. Empty square: a fragment from tetracycline-resistance gene; error bar: standard deviation.

**Figure 4:**
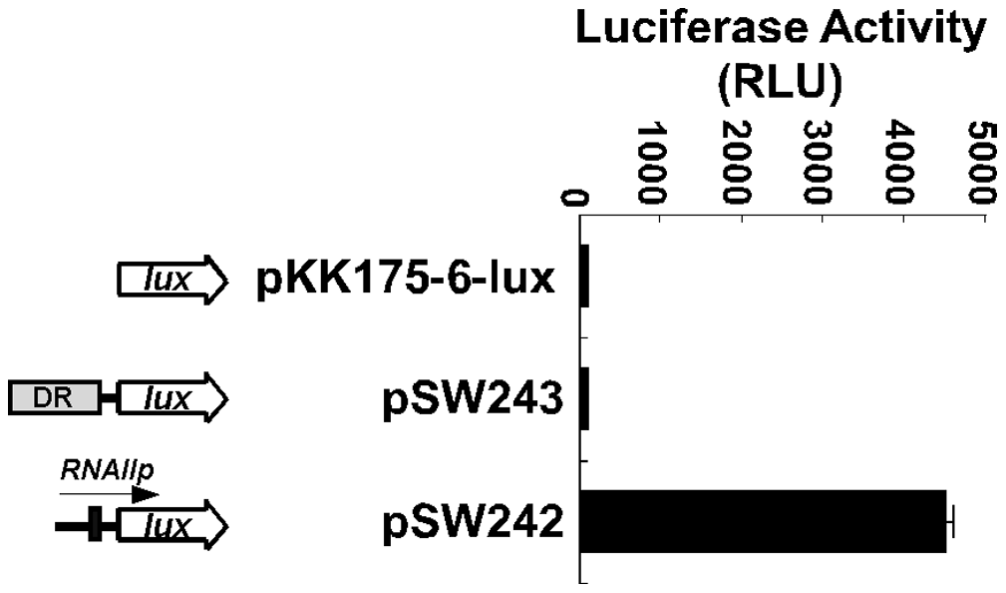
Transcription from DR and *RNAIIp*. Transcriptional fusion was generated by inserting a fragment from nt 1 to 426, which contains *RNAIIp* in pSW201 (pSW242), and a fragment from nt 3356 to 4367 in pSW201, which contains DR (pSW243), into a luciferase reporter plasmid, pKK175-6-lux. Luciferase activity was monitored with a luminometer and presented in relative light units (RLU). Each experiment was performed three times and each sample in the experiment was prepared in duplicate.

As is generally known, RNAI is the *inc* determinant that inhibits the coupling of preprimer RNA to the *oriV* and thereby prevents the replication of ColE1 plasmids. This study posits that deleting the DR region from pSW200 reduces the transcription of the *RNAI* and *preprimer RNA* genes. Restated, in a cell that contains both pSW201 and pSW207, pSW201 expresses more RNAI than does pSW207, overwhelming the preprimer RNA from pSW207 to inhibit the replication of pSW207, which is therefore excluded from the cell. Therefore, in this study, transcriptional fusions were generated to test whether the DR region affected the activities of *RNAIp* and *RNAIIp*, which were determined by measuring the amount of RNA that was transcribed from the promoters by RT-qPCR. Additionally, instead of amplifying the region between *RNAIp* and *RNAIIp*, which has a complex secondary structure and is difficult to amplify by PCR, the regions downstream of the two promoters were replaced with a sequence from a Tc-resistance gene (Fig. 3A). RT-qPCR analysis revealed that deleting the DR regions from pSW261 and pSW263 reduced the amount of RNA that was transcribed from *RNAIIp* and *RNAIp* by 29% and 33%, respectively (Fig. 3B, 3C), revealing that the deletion of the DR region equally reduced the activities of *RNAIp* and *RNAIIp* by about 30%. These results suggest that the repeats promote the transcription of the *RNAI* and *preprimer RNA* genes. This enhancement is not attributed to the transcription from the DR repeats as a transcriptional fusion between the repeat region and *luxAB* (pSW243) yielded only the background level of luciferase activity (Fig. 4), indicating that the DR repeats do not contain a promoter that transcribes the *RNAI* and *preprimer RNA* genes.

### Analysis of RNA Polymerase Bound to 15-bp Repeats

**Figure 5:**
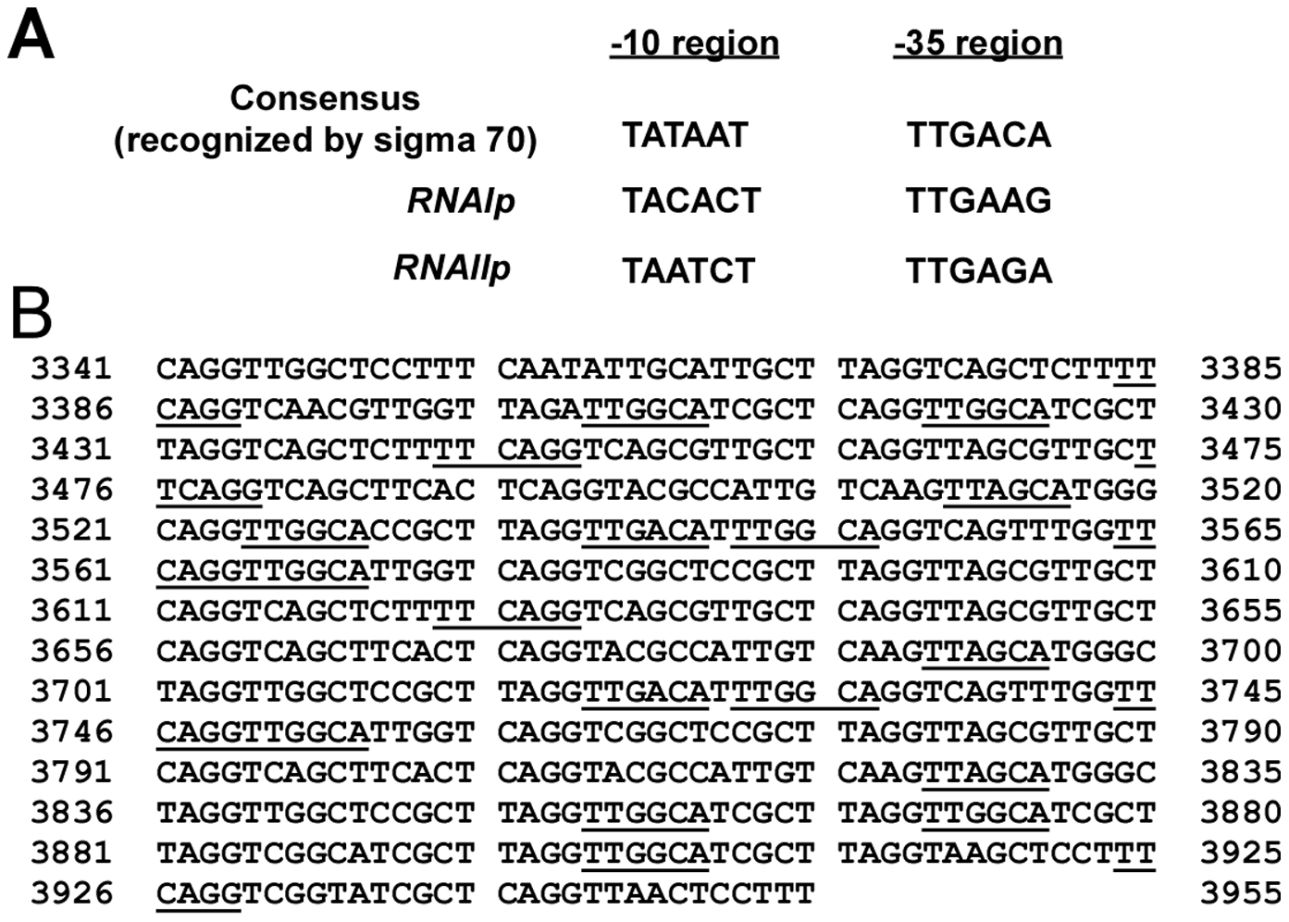
−10 and −35 sequences in *RNAIp*, *RNAIIp*, and DR. (A) Sequences of -10 and -35 boxes in *RNAIp* and *RNAIIp*. (B) Forty-one copies of 15-bp repeats in DR region, from nt 3341 to nt 3955 in pSW200. Sequences that are homologous to the -35 box are underlined.

**Figure 6:**
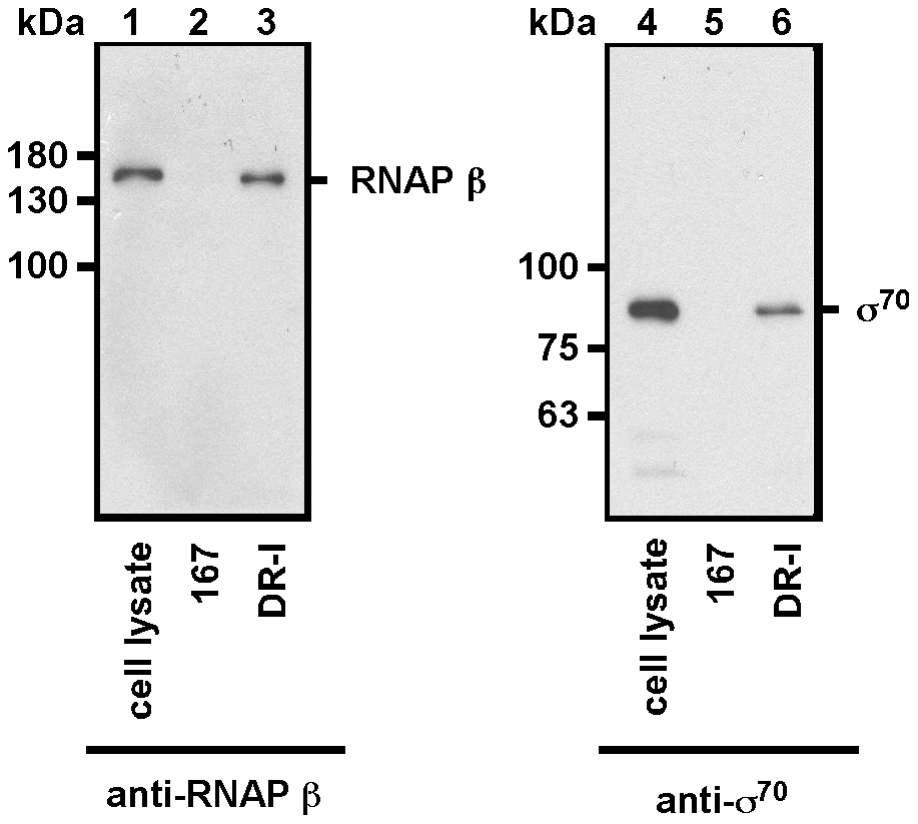
Analysis of proteins that bind to 15-bp repeats. An *E. coli* MG1655 lysate was mixed with a biotin-labeled probe, DR-I, which contained the entire DR region (lanes 3, 6), or probe 167 (lanes 2, 5). Proteins in the cell lysate that was bound to the probes were captured using streptavidin-coated magnetic beads and analyzed by immunoblotting using antibodies against β subunit of RNA polymerase (RNAP β)) (lanes 1–3) and σ^70^ (lanes 4–6). Lanes 1 and 4 were loaded with 0.05% cell lysate.

According to sequence analysis, the repeat region contains sequences resemble that of the -35 box (Fig. 5). To confirm whether the binding of RNA polymerase to the repeats is critical to plasmid competition, a DNA affinity precipitation assay (DAPA) was performed. The lysate from *E. coli* MG1655 was mixed with a biotinylated DNA probe, DR-I, that contained the entire DR region (Fig. 5B). The proteins that were bound to the repeats were analyzed by immunoblotting using antibodies against RNA polymerase β subunit and σ^70^_._ The immunoblot results revealed these two proteins in the lysate (Fig. 6, lanes 1, 4). Meanwhile, the study found the binding of RNA polymerase β subunit and σ^70^ to the DR-I probe (Fig. 6, lanes 3, 6), but not to probe 167, which contained a non-promoter sequence from the *fen* operon (Fig. 6, lanes 2, 5), suggesting the binding of RNA polymerase holoenzyme to DR repeats.

### Binding of RNA Polymerase to DR Region

**Figure 7:**
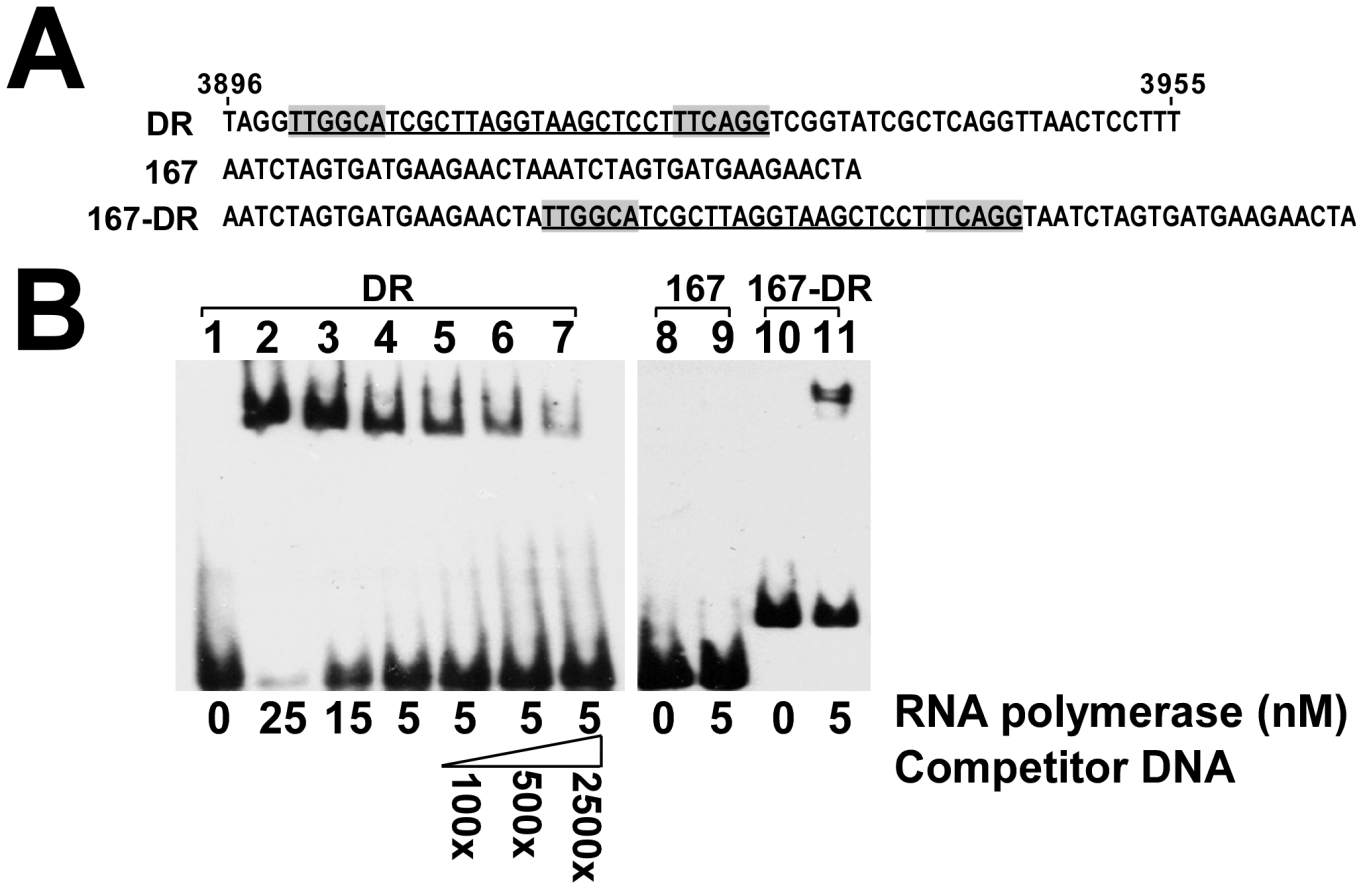
Binding of RNA polymerase to 15-bp repeats. (A) Sequences of DNA probes used in EMSA. DR probe contains four copies of 15-bp repeats from nt 3896 to nt 3955. Sequences highlighted in DR probe resemble **−**35 sequences. Underlined region in DR probe from nt 3900 to nt 3930 was inserted into 167 sequence to yield 167-DR. (B) Purified RNA polymerase holoenzyme (Epicentre) was added to a reaction mixture that contained biotinylated DR probe (lanes 1–7). Unlabeled DR probe was added to compete for binding (lanes 5–7). Probe 167 and 167-DR (lane 8–11) were used to confirm binding of RNA polymerase to repeat region. Protein-DNA complex was separated using a 7% polyacrylamide gel and detected using a LightShift chemiluminescence EMSA kit (Pierce).

The binding of RNA polymerase to the repeat region was confirmed by electrophoretic mobility shift assay (EMSA) using purified enzyme. PAGE revealed that 5–25 nM RNA polymerase shifted 5 nM DR probe (Fig. 7B, lanes 2–4). As expected, adding an unlabeled DR probe to the reaction mixture reduced the binding of RNA polymerase to the biotin-labeled DR probe (Fig. 7B, lanes 5–7). In a negative control, no band shift was observed when 5 nM probe 167 was utilized (Fig. 7B, lanes 8, 9). However, when a segment of the DR sequence from nt 3900 to nt 3930 in pSW200 (Fig. 6B) was inserted into probe 167, as in 167-DR (Fig. 7A), RNA polymerase shifted the probe (Fig. 7B, lanes 10, 11), confirming the binding of RNA polymerase to the DR region.

### Exchange of RNA Polymerase from DR Region to *RNAIp* and *RNAIIp*

**Figure 8:**
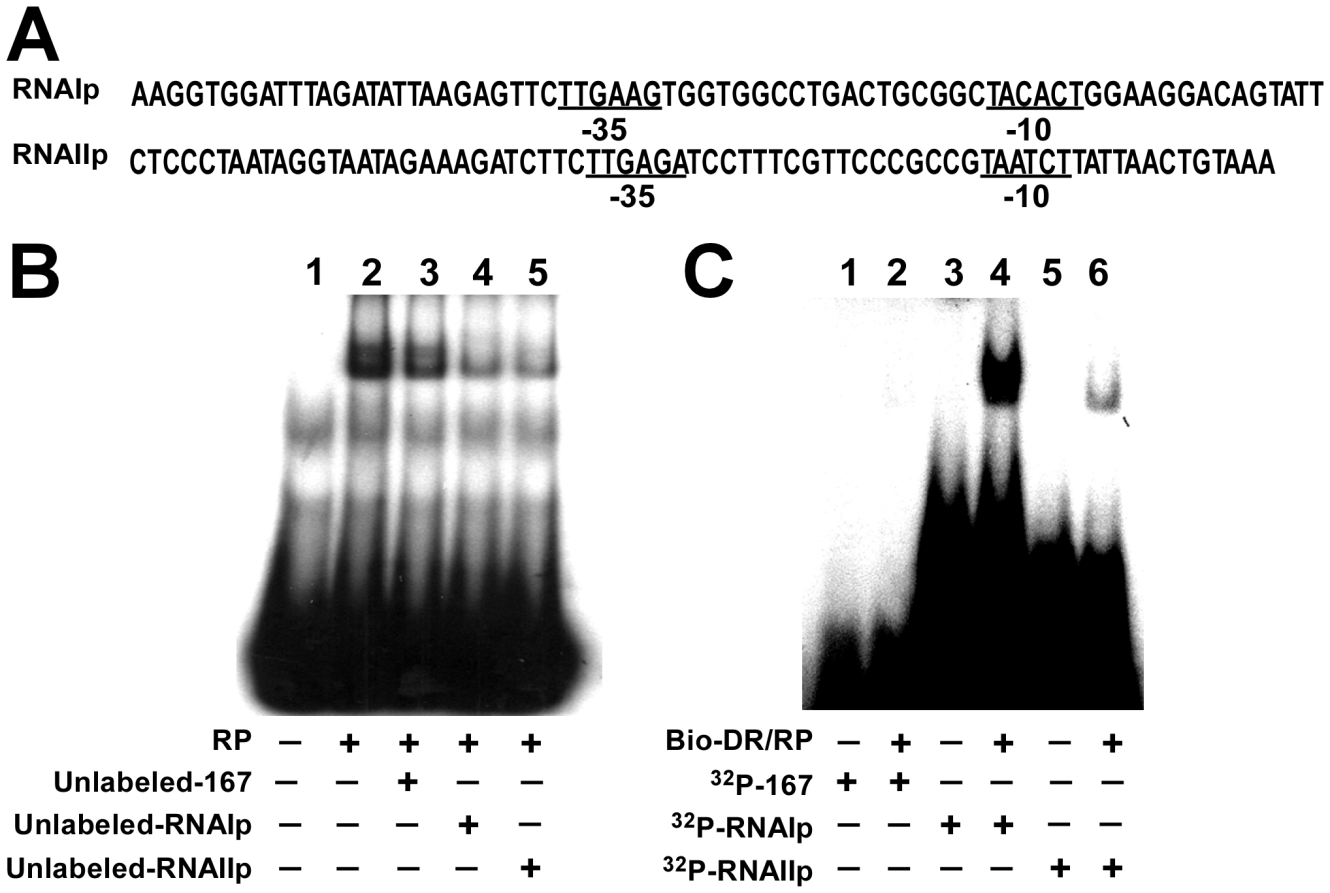
Exchange of RNA polymerase from DR region to *RNAIp* and *RNAIIp*. (A) Sequences of *RNAIp* and *RNAIIp* probes. (B) Purified RNA polymerase (RP) was added to ^32^P-labeled-DR probe (Fig. 7A). Unlabeled *RNAIIp* and *RNAIp* probes, which contained sequences shown in (A), were used to analyze exchange of RNA polymerase from DR region to *RNAIp* and *RNAIIp*. (C) DNA-protein complexes that contained a biotinylated-DR probe (Bio-DR) and RNA polymerase (Bio-DR/RP) were captured using streptavidin-coated magnetic beads. After unbound RNA polymerase had been removed, ^32^P-labeled *RNAIIp* and *RNAIp* probes were added to RNA polymerase-DR complex to analyze exchange of RNA polymerase from repeats to *RNAIp* and *RNAIIp*. Probe 167 was used as a negative control.

Since RNA polymerase binds to the DR region, this study hypothesizes that RNA polymerase was tethered in the repeat region and was subsequently exchanged to *RNAIp* and *RNAIIp*, enhancing the transcription of *RNAI* and *preprimer RNA* genes. Hence, 5 nM RNA polymerase was mixed with 0.1 µM ^32^P-labeled DR probe to enable the binding of the enzyme to the probe. Cold *RNAIp*, *RNAIIp*, and 167 probes (0.1 µM) were then added to the reaction mixture to compete for binding. Although adding probe 167 slightly reduced the amount of RNA polymerase that was bound to the DR probe (Fig. 8B, lane 3), adding *RNAIp* and *RNAIIp* probes substantially reduced the binding (Fig. 8B, lanes 4, 5), indicating that adding *RNAIp* and *RNAIIp* probes to a mixture of RNA polymerase-DR probe complex destabilized the binding of RNA polymerase to DR. Another set of experiments involved adding cold RNA polymerase-DR probe complex (Bio-DR/RP) that had been captured with streptavidin beads to reaction mixtures that contained 0.1 µM ^32^P-labeled *RNAIp*, *RNAIIp* and 167 probes. The results revealed that RNA polymerase-DR complex shifted the *RNAIp* and *RNAIIp* probes (Fig. 8C, lanes 4, 6) but did not shift probe 167 (Fig. 8C, lane 2). Notably, the intensities of the probe bands in the gel seemed high, because of the large amounts of probes that were required to ensure detection of the exchange of RNA polymerase. The results revealed that RNA polymerase bound to the DR probe was exchanged with the *RNAIp* and *RNAIIp* probes.

### Competition of Plasmids of ColE1 Family Caused by pSW200 Repeats

**Figure 9:**
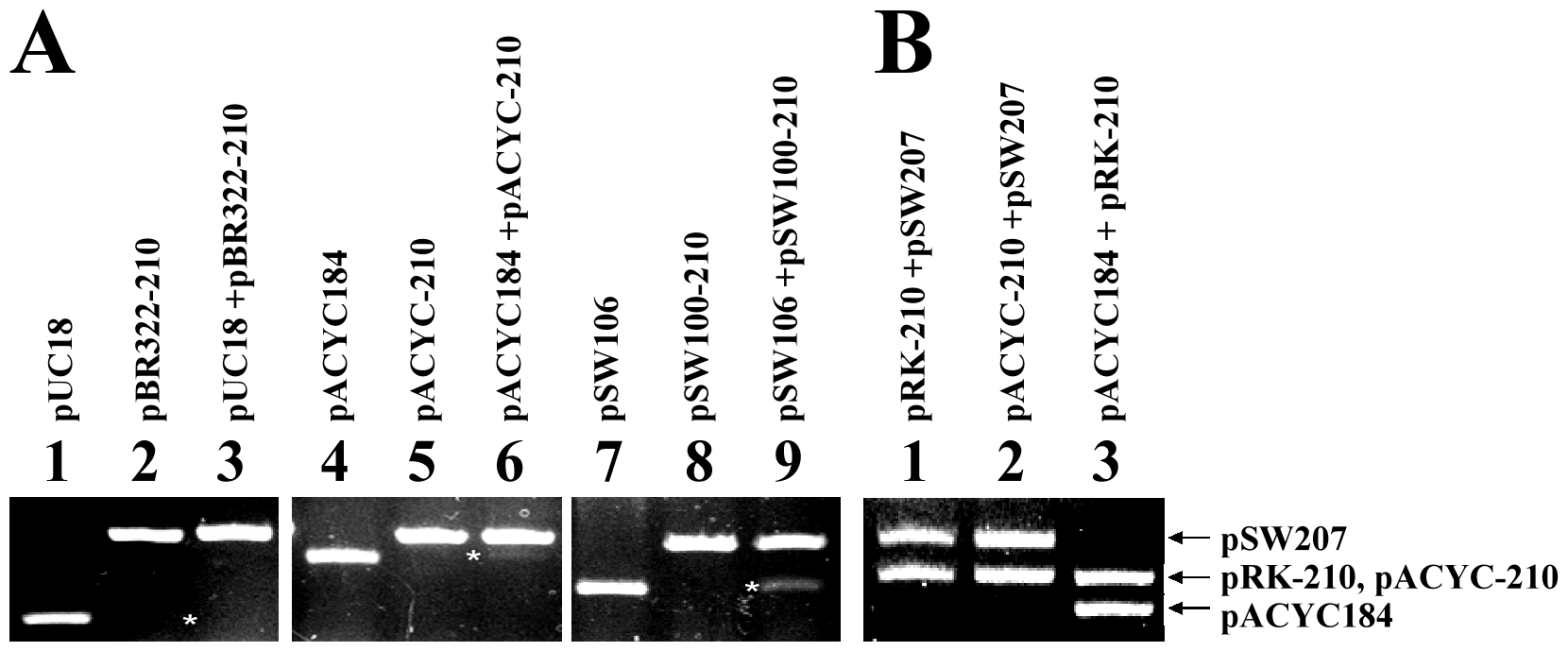
Competition of ColE1-like plasmids by pSW200 repeats. (A) Three plasmids in the ColE1 family that contains DR – pBR322-210, pACYC-210, and pSW100-210 were tested to determine their capacity to destabilize an incompatible plasmid. *E. coli* HB101 was transformed with pUC18 (lane 1), pBR322-210 (lane 2), pACYC184 (lane 4), pACYC-210 (lane 5), pSW106 (lane 7), and pSW100-210 (lane 8). The cells were also cotransformed with pUC18 and pBR322-210 (lane 3), pACYC184 and pACYC-210 (lane 6), and pSW106 and pSW100-210 (lane 9). (B) Plasmids pRK-210, a plasmid that contains an RK2 replicon and DR, and pACYC-210 were tested to determine their ability to destabilize a compatible plasmid. *E. coli* HB101 was cotransformed with pRK-210 and pSW207 (lane 1), pACYC-210 and pSW207 (lane 2), and pRK-210 and pACYC184 (lane 3). Plasmids were isolated using an alkaline lysis method and detected by agarose gel electrophoresis. Asterisks indicate pUC18, pACYC184, and pSW106.

The *Bgl*II-*Acc*I fragment (nt 380 to nt 1178) in pSW210 (Fig. 1B) that contained the replicon was replaced with a pBR322, p15A, or pSW100 replicon to yield pBR322-210, pACYC-210, or pSW100-210, respectively. Gel electrophoresis revealed that although pUC18 had a high copy number [51] when it was maintained as the sole plasmid in *E. coli* HB101 (Fig. 9A, lane 1), the copy number of pUC18 was considerably lower when pBR322-210 was present in the same cell (Fig. 9A, lane 3), showing that pUC18 was lost in the presence of pBR322-210. Meanwhile, pACYC184 and pSW106 were lost in the presence of pACYC-210 and pSW100-210, respectively (Fig. 9A, lanes 6, 9). This study also showed that pRK-210, which contains the RK2 replicon and DR, did not cause the instability of pSW207 and pACYC184 (Fig. 9B, lanes 1, 3). Plasmid pACYC-210 also did not cause the instability of pSW207 (Fig. 9B, lane 2), indicating that the presence of DR in a compatible plasmid does not cause competition. These results demonstrate that the repeats in the DR region from an incompatible plasmid cause competition among the plasmids of the ColE1 family.

## Discussion

Our earlier study revealed that DR is essential to the destabilization of pSW207 by pSW201 in *E. coli* HB101 (Table 2) [35]. This study concludes that the presence of a DR region enhances the transcription of both *RNAI* and *preprimer RNA* genes. RT-qPCR results show that deleting the DR region reduces the activities of both *RNAIp* and *RNAIIp* by about 30%. The results of the RT-qPCR study also suggest that, despite the reduction in transcription, the ratio of RNAI to RNAII that are synthesized by the pSW207 is equal to that of pSW201. This finding may explain why the number of copies of pSW207 is similar to that of pSW201. This study also shows that DR functions in a manner similar to eukaryotic enhancers [52] by promoting transcription from a distance in either orientation, although the ability of DR to enhance transcription is considerably less than that of a typical enhancer.

This study also supports the claim that RNA polymerase binds to the repeats in the DR region and is then exchanged with *RNAIp* and *RNAIIp,* promoting the transcription of the *RNAI* and *preprimer RNA* genes. The first piece of evidence for this claim is that the 15-bp repeats in the DR region contain sequences that resemble that of a -35 box (Fig. 5). Although RNA polymerase typically binds to both -35 and -10 sequences to initiate transcription, RNA polymerase anchors to the -35 region of the promoter to form the first closed complex in the initial stage of transcription [53], [54], suggesting that RNA polymerase binds to the -35-like sequences in the DR region. The second piece of evidence is that the immunoblot analysis revealed the binding of RNA polymerase β’ subunits and σ^70^ to probes that contain the 15-bp repeat sequences (Fig. 6). Finally, an EMSA study revealed that RNA polymerase shifted the probes that contain repeat sequences (Fig. 7B), revealing the binding of RNA polymerase to the DR region.

This study establishes that RNA polymerase that is tethered to DR is exchanged with *RNAIp* and *RNAIIp* since the EMSA results show that adding cold *RNAIp* and *RNAIIp* probes to the reaction mixture considerably reduced the amount of RNA polymerase that was bound to the DR probe (Fig. 8B). Despite the binding of RNA polymerase, DR probable does not act as a promoter, since *luxAB* reporter genes that are inserted downstream of the DR region are not transcribed from DR (Fig. 4). Additionally, a sequence that resembles a -10 sequence is not present at a proper distance from the -35 sequences, suggesting that DR does not have promoter activity. Moreover, the exchange of RNA polymerase from an unlabeled repeat probe becomes evident when ^32^P-labeled probes that contain the sequences of *RNAIp* and *RNAIIp* are added to the reaction mixture (Fig. 8C), suggesting that RNA polymerase that binds to the DR region is exchanged with *RNAIp* and *RNAIIp* to enhance transcription. Finally, pUC18, pACYC184, and pSW100 exhibit the competition phenotype when a DR fragment is present (Fig. 9), suggesting that the DR fragment also affects other ColE1-like plasmids. Furthermore, DR functions only between incompatible plasmids because pRK-210 and pACYC-210 do not cause instability of compatible plasmids (Fig. 9B). Based on the results of this study, a model of the regulation of plasmid competition by DR is proposed. In this model, DR promotes the transcription of *RNAI* and *preprimer RNA* genes of pSW201 by exchanging RNA polymerase that is bound to DR with *RNAIp* and *RNAIIp*. Although pSW207 synthesizes less preprimer RNA than does pSW201, the ratio of RNAI to preprimer RNA remains unchanged, so the copy number of pSW207 is similar to that of pSW201. Meanwhile, the RNAI that is generated by pSW201 efficiently inhibits the coupling of preprimer RNA to *oriV* to decrease the copy number of pSW207, while maintaining the copy number of pSW201 at ten per cell. Therefore, pSW200 uses DR to inhibit the replication of an incoming incompatible plasmid without the DR sequence to prevent curing from the cell by incompatibility. This study elucidates a mechanism by which a plasmid causes the loss of an invading incompatible plasmid to maintain its stability during evolution.
